# Hoffmeister Series Ions Protect Diphtheria Toxoid from Structural Damages at Solvent/Water Interface

**DOI:** 10.3390/ma2030765

**Published:** 2009-07-13

**Authors:** Jocimara A.M. Namur, Célia S Takata, Pedro S. de Araujo, Maria H. Bueno-da-Costa

**Affiliations:** 1 Laboratório de Microesferas e Lipossomas – Centro de Biotecnologia- I. Butantan, Av. Vital Brasil, 1500, 05503-900 - Butantan, São Paulo, SP, Brasil; 2 Divisão de Desenvolvimento Tecnológico e Produção-I. Butantan Av. Vital Brasil, 1500 (05503-900) Butantan, São Paulo, SP, Brasil; E-Mail: celiatakata@butantan.br; 3 I. de Química, (05508-000) Universidade de São Paulo Av. Lineu Prestes 748, São Paulo, SP, Brasil; E-Mails: joci_namur@yahoo.com.br (J.-A.-M.N.); psdarauj@usp.br (P.-S.-D.A.)

**Keywords:** Hoffmeister series ions, protein solubilization, protein stabilization, interaction protein/organic solvent, protein microencapsulation, adjuvant particulate, adjuvant

## Abstract

During the W_1_/O phase (in the W_1_/O/W_2_ process) of protein microencapsulation within poly-lactide-co-glycolide (PLGA), hydrophobic interfaces are expanded where interfacial adsorption occurs followed by protein unfolding and aggregation. Spectroscopic and immunological techniques were used to ascertain the effects of the Hoffmeister series ions on Diphtheria toxoid (Dtxd) stability during the W_1_/O phase. A correlation was established between salts used in aqueous solutions and the changes in Dtxd solubility and conformation. The Dtxd α-helical content was quite stable thus leading to the conclusion that encapsulation was followed by protein aggregation, with minor exposition of hydrophobic residues and a small change at the S-S dihedral angle. Dtxd aggregation is 95% avoided by the chaotropic SCN^-^. This was used to prepare a stable Dtxd and immunologically recognized/PLGA formulation in the presence of 30 mM SNC^-^. The recovery increased by 10.42% or 23.2% when microencapsulation was within the -COOMe or -COOH (12kDa) PLGA, respectively. In conclusion, the aim of this work was achieved, which was to obtain the maximum of Dtxd stability after contact with CH_2_Cl_2_ to begin its PLGA microencapsulation within ideal conditions. This was a technological breakthrough because a simple solution like salt addition avoided heterologous proteins usage.

## 1. Introduction

Protein encapsulation within poly-lactide-co-glycolide (PLGA) microspheres is a simple and straightforward procedure. To date, empirical approaches have been employed for protein stabilization during the first step of the double emulsion process (W_1_/O/W_2_).

In this step high pressures, temperature gradients and shear forces enhance the probability of oxidations to occur, leading to free radicals production and protein damages. When shear forces are considered, their net result is an increase in the organic solvent surface area which triggers protein adsorptions, aggregate precipitations and conformational damages [1,2,3,4,5,6,7,8]. BSA addition to diphtheria toxoid (Dtxd) or to tetanus toxoid (Ttxd) [9,10] was proposed to avoid antigen aggregation. The underlying supposition is that BSA competes with the antigen for the adsorption at the CH_2_Cl_2_/H_2_O interface. Evidently, this can occur. However, it is not reasonable to add heterologous proteins to pharmaceutical formulations intended for human usage. Another proposition was to increase the protein concentration, maintaining the same interfacial CH_2_Cl_2_/H_2_O area. Good results were obtained for carbonic anhydrase and BSA [11], lysozyme [12], and human growth hormone [13]. With insulin [14] the increase in concentration induces gel formation that leads to peptide stabilization. Other ideas were the co-dissolution of PLGA with non ionic surfactants [1,15,16,17,18,19,20,21] and protein chemical modifications with polyethylene glycol (PEG) [22]. It is known, from literature, that the Hoffmeister ions salts are able to change proteins solubilities (both, in water or at interface). Based on these facts it was decided to used them as a simple solution to avoid protein precipitations, aggregations or conformational modifications during the first phase (interfacial contact with CH_2_Cl_2_) of the W/O/W PLGA microsphere preparation. The salt can easily be removed after microsphere precipitation and washing. We discuss the mechanisms by which protein conformation is maintained and damages are avoided.

## 2. Materials and Methods

### 2.1. Materials

Dtxd and anti-diphtheric standard antiserum (developed in horses) were a gift from Instituto Butantan — Divisão de Produção. The immunoconjugates, Triton-X 114, poly-lactide-co-glycolide (PLGA 50:50, 45-75 kDa), dimethylsufoxide (DMSO) and 3,3’,5,5’-tetramethylbenzidine (TMB) were from Polysciences; methylene chloride (CH_2_Cl_2_), polyvinyl alcohol (PVA, MW 49,000) were from Merck and PLGAs (12kDa end terminal free or carboxymethylated) were from Boehringer Ingelheim. All the other reagents were of analytical grade. The following equipments were used: ultraconcentrator (Amicon); Cary 3E UV-Visible spectrophotometer (Varian), F 2000 spectrofluorimeter (Hitachi), spectropolarimeter Jobin Yvon (Spex CD6 Dichrograph Instrument); HPLC (Shimadzu) LC-10VP, equipped with UV detector (model SCL-10AVP); ELISA plate reader (Titerteck) Multiskan MCC/340, homogenizer (Ultra Turrax) T 25 basic IKA Labortechnik, RZR 2051 electronic evaporator (Heidolph) equipped with a tachometer. 

### 2.2. Effects of Hoffmeister series ions on the Dtxd solubility during emulsification with CH_2_Cl_2_

Two mL samples of 5 mM Dtxd in KSCN, MgCl_2_, NaCl or NaH_2_PO_4_ (0, 10, 30, 50, 70, 90, 100 and 150 mM final concentrations) were added to 8 mL CH_2_Cl_2_. After 2 minutes of emulsification at 24,000 rpm in the Ultra Turrax, samples were centrifuged during 15 minutes at 18,154 g. The protein contents of the aqueous phases were measured by the absorbance at 269 nm (24) or by HPLC. The soluble fractions were analyzed by CD, fluorescence and ELISA.

### 2.3. Circular dichroism

The soluble Dtxd fractions in different salts at different concentrations were analyzed using a 0.1 cm path length cuvette in a Jobin Yvon spectropolarimeter. 

### 2.4. Fluorescence

The soluble Dtxd fractions in different salts concentrations were analyzed using quartz cuvettes in a Hitachi F 2000 fluorimeter. The samples were excited at 269 nm and the fluorescence emission measured between 280 and 480 nm.

### 2.5. ELISA

Dtxd soluble samples were added to ELISA plates and after two hours at 37 °C, they were blocked with 10% skim milk. After 30 min, the standard anti-Dtxd specific sera, developed in horses, was added to the wells. The conjugate (anti-horse whole IgG conjugate with peroxidase) was added 30 min later, and, after a further 30 min, the substrate was added. After 15 min at room temperature, the reaction was stopped with 1 M H_2_SO_4_. The absorbance was automatically measured at 450 nm in a Titertek Multiskan MCC/340. *Antibody titters* are the reciprocal serum dilution factor giving an absorbance value of 20% of the saturation value.

### 2.6. Dtxd encapsulation within PLGA in the presence of KSCN

The PLGA particles were prepared using the water in oil/in water [(W_1_/O)/W_2_] double emulsification solvent evaporation method. Briefly, under strong agitation, 125 µL of PBS containing 30 mM KSCN were added to 200 mg of PLGA (12 kDa, end terminal free or carboxymethylated) previously dissolved in 2 mL of CH_2_CL_2_. This mixture was then emulsified at 24,000 rpm for 2 min in an Ultra Turrax emulsifier. The emulsion was quickly added to 40 mL of 0.5% PVA and submitted to emulsification at 19,000 rpm for 2 min. The solvent was evaporated by gentle stirring for 3 hours at 1,000 rpm in a Heidolph^®^ RZR 2051 helix evaporator. The microspheres were collected by centrifugation for 10 min. at 2,000 g, rinsed with water three times and then resuspended with 2 mL of 0.1% PVA, freeze-dried for 24 hours and stored at -20 °C. The encapsulation efficiency was calculated measuring non-encapsulated protein in the supernatant.

## 3. Results

**Figure 1:**
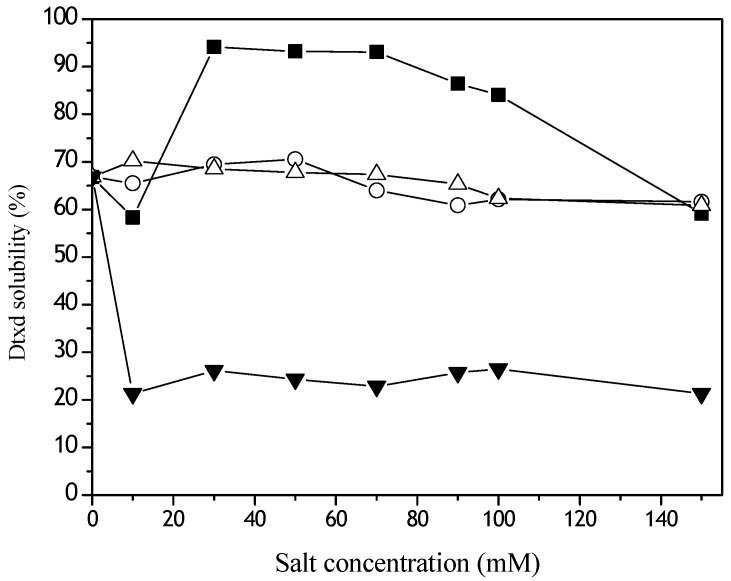
Effects of Hoffmeister series ions on Dtxd recovery during emulsification with CH_2_Cl_2_. The kosmotropics NaHPO_4_ (o), NaCl (∆) or the chaotropics KSNC (■) and MgCl_2_ (▼) were added to the Dtxd water solutions and emulsified in the presence of CH_2_Cl_2_ at 24000 rpm. The soluble protein was measured by HPLC.

The Dtxd solubility was enormously affected by the emulsification both in the presence of CH_2_Cl_2_ and in the absence of salts (Figure 1), when 33% of the molecule precipitated. The kosmotropics NaH_2_PO_4_ and NaCl, practically did not alter protein recovery in all concentrations studied. It was interesting to note that the chaotropic MgCl_2_ precipitated at least 80% of Dtxd in all concentrations used. In contrast, the other chaotropic, KSCN solubilized Dtxd and 95% of the protein was recovered after emulsification in the presence of the organic solvent (Figure 1, 30 – 70 mM KSCN).

**Figure 2:**
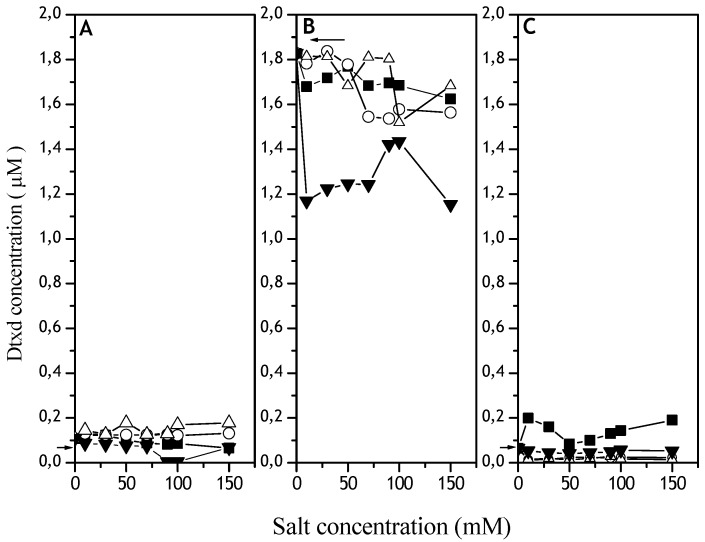
Effect of Hoffmeister series ions in different Dtxd molecular species after emulsification in the presence of CH_2_Cl_2_. The kosmotropics NaHPO_4_ (o) and NaCl (∆) or the chaotropics KSNC (■) and MgCl_2_ (▼) were added to the Dtxd water solutions and emulsified in the presence of CH_2_Cl_2_ at 24,000 rpm. **A.** Dtxd dimmer, **B.** Dtxd monomer and **C.** Fragments A and B. Controls: Dtxd emulsified without salt and methylene chloride (arrows in the pictures). The soluble Dtxd molecular species fractions were analyzed by HPLC gel filtration (QC-PACK GFC 300 column - 7.8 mm X 15 cm) previously equilibrated with PBS, at 20 °C. Injection and elution flows were 0.6 mL/min.

In order to know the nature and extent of these Dtxd losses, the soluble Dtxd fractions after emulsification in the presence of salts were applied on a gel filtration HPLC column (Figure 2). The dimer (2 x Dtxd) concentration was practically the same in all salt concentrations used (Figure 2A). There is a large reduction in Dtxd monomers when the emulsification was done in the presence of MgCl_2_ (Figure 2B). The increase in FA and FB (Fragments A and B) occurred in concert with the decrease of Dtxd monomers when Dtxd was emulsified in the presence of SCN^-^, suggesting a significant Dtxd monomer hydrolysis. The decrease in monomers in the presence of Mg^2+^ and PO_4_^2-^ (Figure 2B) did not coincide with the dimer, FA or FB increases (Figure 2A). Probably, in these situations insoluble aggregates (containing more than 2 Dtxd monomers) could be formed, since the Dtxd dimer was still soluble (Figure 2A).

**Figure 3:**
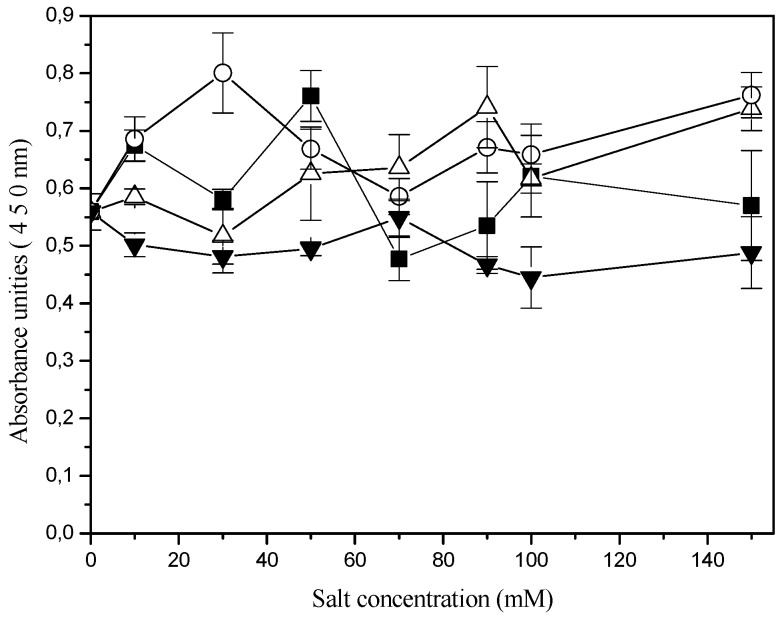
Immunological identity of the soluble Dtxd fractions. The kosmotropics NaHPO_4_ (o) and NaCl (∆) or the chaotropics KSNC (■) and MgCl_2_ (▼) were added to the Dtxd solution in water and emulsified in the presence of CH_2_Cl_2_ at 24,000 rpm. The soluble fractions were analyzed by ELISA. Control: untreated protein with an absorbance of 0.52 absorbance units.

The lesser immunological identity of these fractions was observed when the emulsification was done in the presence of MgCl_2_ (Figure 3). The other salts, practically, did not alter the immunological identity of the soluble Dtxd fractions (Figure 3).

**Figure 4:**
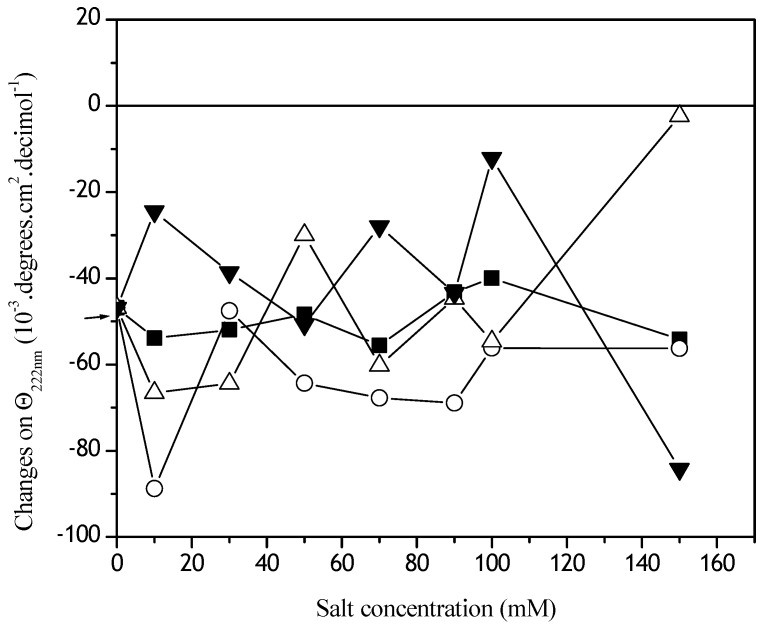
Effect of Hoffmeister series ions concentrations on θ_222nm_ Dtxd. Soluble samples of 7.36 mM Dtxd were obtained after emulsification in the presence of CH_2_Cl_2_ and the kosmotropics NaHPO_4_ (o) and NaCl (∆) or the chaotropics KSNC (■) and MgCl_2_ (▼) and analyzed by CD spectroscopy.

**Figure 5:**
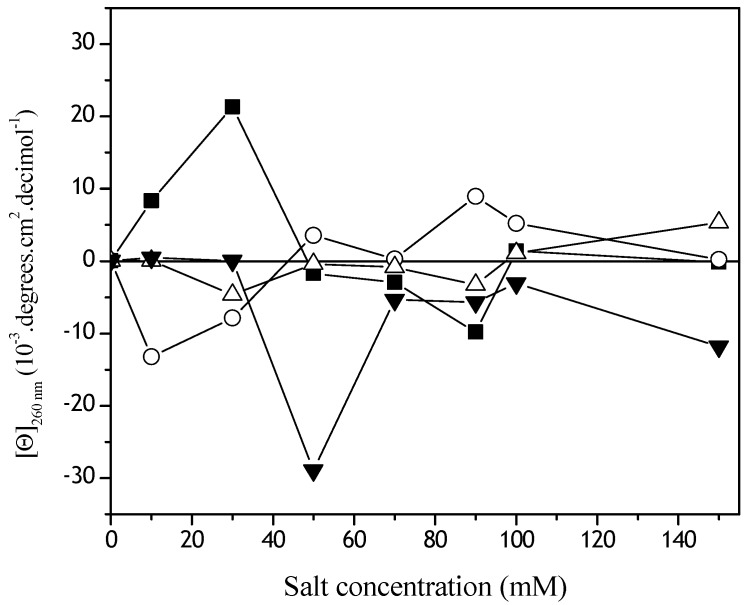
Effect of Hoffmeister series ions concentrations on θ_260nm_ Dtxd. Soluble samples of 7.36 mM Dtxd were obtained after emulsification in the presence of CH_2_Cl_2_ and the kosmotropics NaHPO_4_ (o) and NaCl (∆) or the chaotropics KSNC (■) and MgCl_2_ (▼) and CD spectra were recorded (cuvettes of 0.1 cm path length).

The extent of these conformational damages was analyzed by circular dichroism (Figure 4). Changes in the α−helical content were observed at θ_222nm_ (Figure 4) and for S-S dihedral angle conformations, at θ_260nm_ (Figure 5). The α−helical content in the presence of NaH_2_PO_4_ increased in all the concentrations studied (Figure 4). Oscillations on θ_222nm_ in the lower NaCl concentration were observed. The biggest negative Cotton effect was observed in the Dtxd emulsified in the presence of MgCl_2_. In the presence of KSCN the Dtxd α−helical content remained stable (Figure 4).

**Scheme 1:**
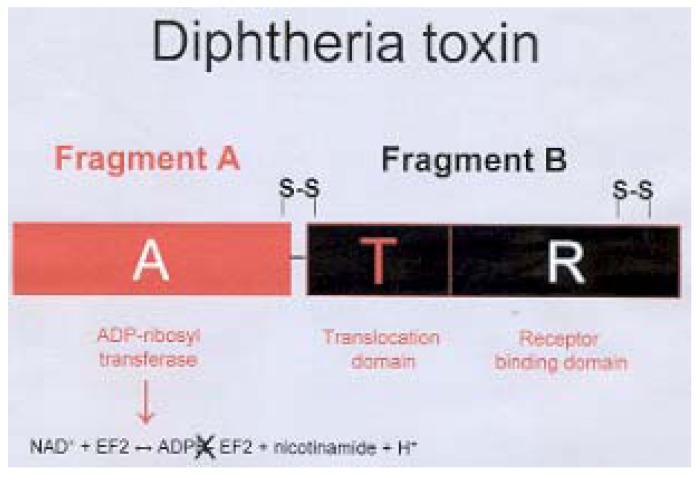
Representation of Dtxd (adapted from [23]).

To facilitate the discussion, a schematic representation of Dtxd is shown in Scheme 1. It is important to note that diphtheria toxin has two S-S bridges (C_186_-C_201_ and C_461_-C_471_). One of them is an inter-peptide linkage (FA-FB, C_186_-C_201_) and the other is an intra-peptide linkage (FB, C_461_-C_471_). The dihedral S-S angle (linkage between the FA and FB) suffered conformational alterations during the simulation of the first emulsification during the microsphere preparation method (Figure 5). Probably, the observed dihedral changes are related with the C_186_-C_201_ bridge because of the steric hindrance and constraints on the other S-S linkage (C_461_-C_471_). It is known that angular dihedral variations ≥ 120° are related with a negative Cotton effect. In contrast, angular dihedral variations ≤ 60° induce positive Cotton effects [25,26].

The lowest S-S dihedral conformational change was observed when Dtxd was emulsified in the presence of NaCl (Figure 5). From 10-40 mM NaH_2_PO_4_ the S-S dihedral change was ≥ 120° and above 50 mM NaH_2_PO_4_ remained ≤ 60°. The biggest Dtxd S-S variations were observed in the presence of MgCl_2_. In the presence of MgCl_2_, the Dtxd opened its structure (the S-S changed from ≤ 60°, a positive Cotton effect, to ≥ 120° a negative Cotton effect). Between 50 and 70 mM KSCN, the S-S dihedral Dtxd angle also changed its conformation, but the variations were close to zero and corresponded to a closed conformation. 

**Figure 6:**
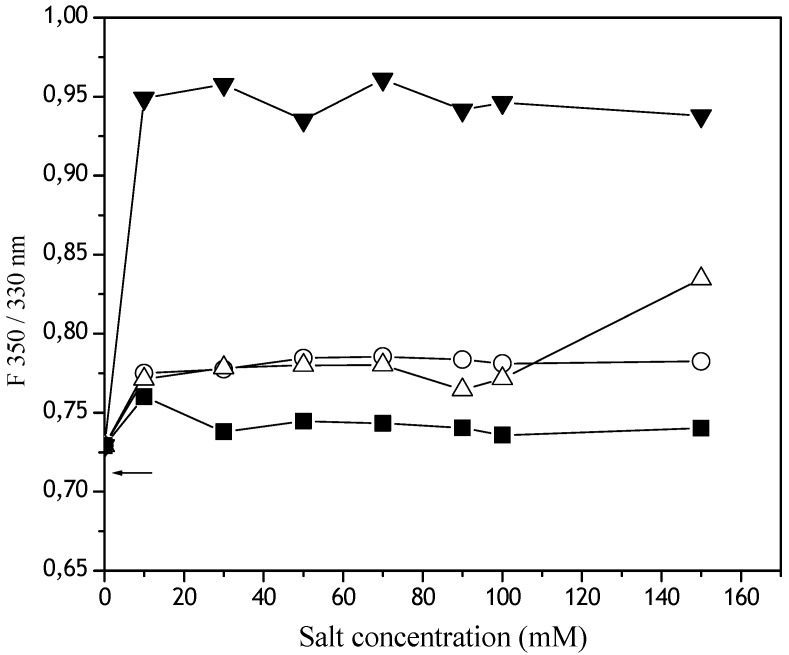
Effect of Hoffmeister series ions on Dtxd intrinsic fluorescence. Soluble samples of 7.36 mM Dtxd were obtained after its emulsification in the presence of CH_2_Cl_2_ and the kosmotropics NaHPO_4_ (o) and NaCl (∆) or the chaotropics KSNC (■) and MgCl_2_ (▼) and the fluorescence spectra were recorded.

It was observed in the fluorescence spectra that all MgCl_2_ concentrations studied produced the highest increase in the F350 nm/F330 nm ratio (that corresponds to the degree of tryptophan, W, exposition to the media). The NaCl and the NaH_2_PO_4_ induced an intermediate W exposition. The lowest W exposition was observed in the presence of KSCN (Figure 6).

Taking all the previous results into consideration, Dtxd microencapsulations within MS-PLGA in the presence of KSCN were performed and under this condition, the encapsulation efficiency raised from 53.84% to 64.26 (when the microencapsulation of Dtxd was done within the end methylated PLGA of 12 kDa) and 23.2% (from 46.41% to 69.63%) within the free –COOH PLGA (12 kDa). 

## 4. Discussion

It was verified in this work that simple molecules like Hoffmeister salts could be the best solution to protect the Dtxd protein from aggregation and conformational damages during the emulsification with CH_2_Cl_2_. Salts are easier to remove than exogenous proteins. The problem we faced was solved because high quantities of Dtxd were solubilized by SCN^-^ and consequently increased the encapsulation efficiency.

The enhancement of Dtxd protein solubility in the presence of SCN^-^ contrasted with other literature results, where lisozyme was precipitated in the presence of this chaotropic [26]. When the solvent is changed (ethyl acetate, for example) the SCN^-^ decreased Dtxd solubility [27], meaning that the protein partition depends on several factors like dielectric constant.

The problem approached in this work was not simple neither trivial: a ternary system composed by the protein Dtxd, the water and the organic solvent where a fourth element would be added - the salt, under a 24,000 rpm agitation! It would be an oversimplification to opt for one of the theories [28,29,30,31] to explain the Hoffmeister effect on protein solubility. The protein solubility change [30,31] in the presence of kosmotropic salts which stabilize solutes, by increasing the order of water or in the presence of chaotropic salts which create weaker hydrogen bonding thus decreasing the order of water and increasing its surface tension. Nowadays it is known that the Hoffmeister series mechanism of action is related to ion specific phenomena [29]. But, changes on water structure after salt additions do not explain the ion specific phenomenon [28,32]. Here the chaotropics SCN^-^ and Mg^2+^ were used. In addition to their charge difference it is known that SCN^-^ is poorly hydrated and Mg^2+^ is strongly hydrated [28,32]. The Dtxd was in water, pH 6.5, and containing salt in a condition of 2 pH units above its pI [23] with a net negative charge. Therefore, it is reasonable to assume that an ionic interaction between Dtxd (negatively charged) and Mg^2+^ did not favor protein solubility. In this salt condition the protein could precipitate after organic solvent contact, maximized by the agitation at 24000 rpm. So, here, the Hoffmeister series would be understood as an ion specific phenomenon [28,33]. The kosmotropic salts, NaCl and NaH_2_PO_4_ did not alter the Dtxd aggregation caused by agitation. The aggregation caused by the presence of CH_2_Cl_2_ was not associated to the increase in Dtxd dimmer (the dimmer is soluble) but with the formation of large aggregates. Dtxd was not hydrolyzed in FA and FB. The Dtxd soluble fraction immunologically recognized was lost after emulsification in the presence of MgCl_2_. This Dtxd not immunologically recognized conformation corresponded to a protein more folded and more exposed to the environment. This exposed protein showed its hydrophobic residues to the media. This fact corroborates the expressed hypothesis that Mg^2+^ interacted strongly with Dtxd. The SNC^-^ protected Dtxd from unfolding, S-S dihedral opening and immunological damages. The Dtxd solubility increased 95% in the presence of SNC^-^, corresponding to a molecule in its native conformation. This technological gain was used to prepare a Dtxd/PLGA formulation in the presence of 30 mM SNC^-^. The recovery increased in 10.42% (from 53.84% to 64.26%) the microencapsulation of Dtxd within the end methylated PLGA (12 kDa) and 23.2% (from 46.41% to 69.63%) within the free –COOH PLGA (12 kDa). 

## 5. Conclusions

In conclusion, the aim of this work was achieved, which was to obtain the maximum protein solubility after emulsification in the presence of CH_2_Cl_2_ to begin its PLGA microencapsulation within ideal conditions. This was a technological breakthrough because a simple solution such as salt addition avoided the usage of heterologous protein. 
